# Threatened Respiratory Compromise in the Setting of Isolated Angioedema

**DOI:** 10.5811/cpcem.2018.9.39548

**Published:** 2018-09-28

**Authors:** Graham S. Stephenson, Shahram Lotfipour, Shadi Lahham

**Affiliations:** * University of California, Irvine, School of Medicine, Irvine, California; † University of California, Irvine, Department of Emergency Medicine, Orange, California

## Abstract

Isolated angioedema of the uvula, or Quincke’s disease, is a rare condition that can cause respiratory compromise. Although typically self-limiting, episodes of angioedema may require prompt therapy to prevent obstruction of the proximal airway. In this case report we review the appropriate steps for initial evaluation of patients with suspected angioedema, primary etiologies, and appropriate initial therapy.

## INTRODUCTION

We present a patient with isolated angioedema of the uvula via a likely mechanism of drug-induced histaminergic release vs. direct thermal insult. Although histaminergic angioedema rarely causes respiratory compromise, we report here how mechanical obstruction can create a life-threatening emergency even without associated signs of anaphylaxis. The patient responded well to antihistamine medications and steroids, and was discharged after resolution of symptoms.

## CASE REPORT

A 55-year-old male presented to the emergency department (ED) complaining of one hour of difficulty breathing that woke him from sleep. Symptoms worsened when lying down on his left side. He endorsed a mild sore throat that was felt in the oropharynx; however, he spoke in a normal tone of voice and denied any difficulty swallowing, fever, nausea, vomiting, diarrhea, cough, or previous neck surgery or radiation. He denied history of food allergies, drug allergies, or reaction to toxic insults. He denied any recent changes to diet or travel. He was employed as a mechanic but denied prolonged exposure to exhaust or working without appropriate protective equipment. He admitted to frequent methamphetamine smoking, most recently the evening before presenting to the ED. He denied any current medications, previous exposure to angiotensin-converting-enzyme (ACE) inhibitors, or previous adverse reaction to nonsteroidal anti-inflammatory drugs (NSAIDs). He denied previous diagnosis of lymphoproliferative disorders or family history of angioedema.

Upon arrival to the ED, the patient had the following vital signs: blood pressure 141/93 millimeters of mercury, temperature 98.8° F, heart rate 86 beats per minute, respiratory rate 16 breaths per minute, and oxygen saturation 99% on room air. On physical exam his lungs were clear to auscultation bilaterally, without vesicular breath sounds and no evidence of stridor or wheezing. Oropharyngeal exam did not reveal any significant erythema; however, the patient’s Mallampati score of 4 obstructed sufficient visualization of the posterior oropharynx. Using a tongue depressor, we observed an erythematous and edematous uvula (Image). No tonsillar hypertrophy or exudates were observed. Given the patient’s ability to speak in full sentences with normal oxygen saturation on room air, he was not deemed an appropriate candidate for intubation despite continued complaint of shortness of breath.

Laboratory values of complete blood count and complete metabolic panel were unremarkable, reducing the likelihood of infectious etiology. Due to the patient’s discomfort and the likelihood of inflammation vs. angioedema etiology of complaint, he was treated with 60 milligrams (mg) of methylprednisolone, 25 mg of diphenhydramine, and 20 mg of famotidine. The patient markedly improved within several hours and reported that he felt “100% better.” He was discharged with a prescription of 20 mg prednisone once daily for a three-day course. Strict return precautions were given and he was instructed to follow up with his primary care physician.

## DISCUSSION

Angioedema is a self-limiting condition that results from fluid extravasation into subcutaneous or submucosal tissues. Angioedema typically has a rapid onset with preferential involvement of the face, lips, larynx, and bowels.^1^ This case presentation of uvular angioedema, or Quincke’s disease, is an example of an uncommon yet potentially life-threatening condition that may be seen in the ED. Specifically, this patient presented with symptoms of a histaminergic angioedema with threatened respiratory compromise. Frequent triggers of this complication are infection, calcium channel blockers, or eosinophilia. In some cases, drug use has been attributed as the inciting agent, which may have been the case in our patient.

For patients with suspected angioedema or isolated angioedema a detailed medication and allergy history is critical.^2^ Reported family history of subcutaneous swelling or patients complaining of recurrent bouts of angioedema with abdominal pain should raise suspicion for hereditary angioedema or acquired C1-inhibitor deficiency. A basic array of laboratory tests is necessary for confident diagnosis, including complete blood count, chemistry panel with liver function tests, and inflammatory markers.^3^

The primary etiologies of angioedema are mast cell or bradykinin mediated.^3^ Mast cell-mediated causes, such as allergic reaction or adverse reaction to aspirin or NSAIDS, typically present with acute onset and associated urticarial rash, generalized pruritus, bronchospasm, and hypotension. In certain cases, angioedema may be histaminergic in origin but not associated with mast-cell degranulation.^4^ In these events there is usually no associated urticarial rash or respiratory compromise. If the airway is patent without concern for compromise then antihistamine and glucocorticoid administration is the first-line therapy. Airway compromise or signs of anaphylaxis should instigate immediate intramuscular epinephrine with the aforementioned therapies. In cases without a clear inciting agent causing angioedema, reports of high doses of antihistamines have been helpful (up to 200 mg of diphenhydramine).^5^ For severe and refractory cases there are successful treatments with dapsone, icatibant, and rituximab found in the literature.^6^,^7^,^8^

**CPC-EM Capsule**

What do we already know about this clinical entity?

Uvular angioedema (Quincke’s disease) is a well-established phenomenon within the current literature.

What makes this presentation of disease reportable?

This report shows how Quincke’s disease may present without a clear trigger or systemic signs. Additionally, it highlights the importance of the physical exam.

What is the major learning point?

This case delineates the signs and symptoms in patients presenting with angioedema, the appropriate first-line diagnostic and therapeutic approach, and a strategy to help identify the underlying etiology.

How might this improve emergency medicine practice?

This report seeks to reinforce the diagnostic and therapeutic approach to treating patients with Quincke’s disease in the emergency department.

Bradykinin-induced angioedema is typically not associated with urticarial rash, pruritus, or bronchospasm and develops over a period of days. ACE inhibitors and fibrinolytic therapies are common aggravating agents. Inherited and acquired angioedema causes also fall into this class. If family history is concerning, quantifying complement component 4 may be prudent. For hereditary angioedema with C1-inhibitor deficiency or acquired C1-inhibitor deficiency, consider on-demand treatment with icatibant, ecallantide, or recombinant C1 inhibitor. Fresh frozen plasma is a second-line therapy if none of the above is available.^9^

## CONCLUSION

In the ED, respiratory distress is a frequently encountered chief complaint that warrants immediate evaluation for the underlying cause. This case of isolated uvular angioedema – an uncommon yet potentially life-threatening condition – highlights the breadth of possibilities that need to be considered when approaching a non-specific patient complaint. Here we have outlined the appropriate history, physical exam, critical laboratory values, and therapies that emergency physicians should be familiar with to manage isolated angioedema.

Documented patient informed consent and/or Institutional Review Board approval has been obtained and filed for publication of this case report.

## Figures and Tables

**Image:**
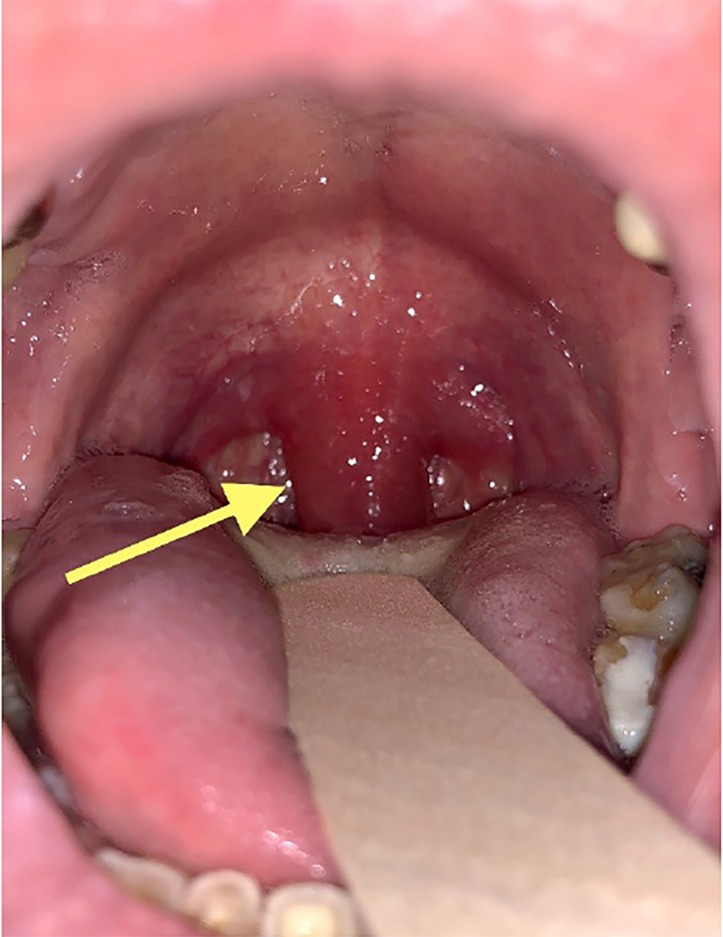
Picture of oropharynx during primary evaluation of patient demonstrating isolated uvular angioedema (arrow).
